# Management and Treatment of Patients With Obstructive Sleep Apnea Using an Intelligent Monitoring System Based on Machine Learning Aiming to Improve Continuous Positive Airway Pressure Treatment Compliance: Randomized Controlled Trial

**DOI:** 10.2196/24072

**Published:** 2021-10-18

**Authors:** Cecilia Turino, Ivan D Benítez, Xavier Rafael-Palou, Ana Mayoral, Alejandro Lopera, Lydia Pascual, Rafaela Vaca, Anunciación Cortijo, Anna Moncusí-Moix, Mireia Dalmases, Eloisa Vargiu, Jordi Blanco, Ferran Barbé, Jordi de Batlle

**Affiliations:** 1 Group of Translational Research in Respiratory Medicine Institut de Recerca Biomèdica de Lleida (IRBLleida) Hospital Universitari Arnau de Vilanova and Santa Maria Lleida Spain; 2 Centro de Investigación Biomédica en Red de Enfermedades Respiratorias (CIBERES) Madrid Spain; 3 eHealth Unit Eurecat Centre Tecnòlogic de Catalunya Barcelona Spain; 4 Barcelona Centre for New Medical Technologies (BCN Medtech) Department of Information and Communication Technologies Universitat Pompeu Fabra Barcelona Spain; 5 Oxigen salud Barcelona Spain

**Keywords:** obstructive sleep apnea, continuous positive airway pressure, patient compliance, remote monitoring, machine learning

## Abstract

**Background:**

Continuous positive airway pressure (CPAP) is an effective treatment for obstructive sleep apnea (OSA), but treatment compliance is often unsatisfactory.

**Objective:**

The aim of this study was to assess the effectiveness and cost-effectiveness of an intelligent monitoring system for improving CPAP compliance.

**Methods:**

This is a prospective, open label, parallel, randomized controlled trial including 60 newly diagnosed patients with OSA requiring CPAP (Apnea–Hypopnea Index [AHI] >15) from Lleida, Spain. Participants were randomized (1:1) to standard management or the MiSAOS intelligent monitoring system, involving (1) early compliance detection, thus providing measures of patient’s CPAP compliance from the very first days of usage; (2) machine learning–based prediction of midterm future CPAP compliance; and (3) rule-based recommendations for the patient (app) and care team. Clinical and anthropometric variables, daytime sleepiness, and quality of life were recorded at baseline and after 6 months, together with patient’s compliance, satisfaction, and health care costs.

**Results:**

Randomized patients had a mean age of 57 (SD 11) years, mean AHI of 50 (SD 27), and 13% (8/60) were women. Patients in the intervention arm had a mean (95% CI) of 1.14 (0.04-2.23) hours/day higher adjusted CPAP compliance than controls (*P*=.047). Patients’ satisfaction was excellent in both arms, and up to 88% (15/17) of intervention patients reported willingness to keep using the MiSAOS app in the future. No significant differences were found in costs (control: mean €90.2 (SD 53.14) (US $105.76 [SD 62.31]); intervention: mean €96.2 (SD 62.13) (US $112.70 [SD 72.85]); *P*=.70; €1=US $1.17 was considered throughout). Overall costs combined with results on compliance demonstrated cost-effectiveness in a bootstrap-based simulation analysis.

**Conclusions:**

A machine learning–based intelligent monitoring system increased daily compliance, reported excellent patient satisfaction similar to that reported in usual care, and did not incur in a substantial increase in costs, thus proving cost-effectiveness. This study supports the implementation of intelligent eHealth frameworks for the management of patients with CPAP-treated OSA and confirms the value of patients’ empowerment in the management of chronic diseases.

**Trial Registration:**

ClinicalTrials.gov NCT03116958; https://clinicaltrials.gov/ct2/show/NCT03116958

## Introduction

Obstructive sleep apnea (OSA) is the most prevalent sleep-disordered breathing condition, affecting 15%-30% of adults in Western countries [1]. It is characterized by repetitive episodes of airways collapse during sleep, causing sleep fragmentation, intermittent hypoxia, and daytime somnolence. OSA has been associated with increased morbidity and mortality, and has an impact on quality of life [2]. In this sense, increased inflammation, oxidative stress, sympathetic activation, and hypercoagulability are the main mechanisms associating OSA with hypertension; cancer; and cardiovascular, cerebrovascular, and metabolic diseases [2].

Nocturnal continuous positive airway pressure (CPAP), preventing upper airway collapse during sleep, is the treatment of choice for patients with symptomatic OSA [3]. A satisfactory CPAP compliance (≥4 hours/day) improves daytime sleepiness and overall quality of life; reduces OSA severity markers, such as the Apnea–Hypopnea Index (AHI); moderately decreases arterial blood pressure (BP), mainly in patients with resistant hypertension [3,4]; and contributes to preventing the onset of newly diagnosed hypertension [5]. Compliance is, therefore, essential for the efficacy of CPAP treatment and its optimization is an important aspect of patient management. However, up to one-third of patients underuse or even discontinue CPAP [6-8], mostly because of treatment-related side effects such as machine noise, pressure intolerance, mask displacement, or claustrophobia [9]. In this sense, issues hampering CPAP compliance during the first months of treatment are likely to have a significant impact on long-term CPAP compliance [10]. Therefore, there is a need to implement effective strategies for the promotion of CPAP compliance, especially during the first months of treatment.

So far, interventions tackling CPAP compliance, including novel educational and supportive or therapeutic strategies, have reported low to moderate evidence of success [11,12]. By contrast, when these strategies are wrapped up in comprehensive packages making use of information and communication technologies (eHealth) and targeting the initial months after CPAP prescription, the potential for success can be significantly enhanced [12-14]. In this scenario, and within the frame of the MiSAOS project, an internet of things–based intelligent monitoring system relying on machine learning [15] was developed in Catalonia, Spain, with a fourfold goal: (1) predicting patient’s potential early CPAP compliance; (2) providing real-time monitoring of patient’s CPAP compliance, informing both the patient and the care team, and granting decision support; (3) empowering the patient by means of feedback and recommendations; and (4) reducing patient’s overall management costs. This study compares, in terms of effectiveness and cost-effectiveness, the MiSAOS intelligent monitoring system model, based on early compliance detection, compliance prediction, and rule-based recommendations, with the usual care provided to patients using CPAP in the region of Lleida, Catalonia.

## Methods

### Study Design

This is a prospective, open label, parallel, randomized controlled trial comparing the MiSAOS management model with care as usual for a duration of 6 months after CPAP prescription (ClinicalTrials.gov NCT03116958). The study was conducted from November 2016 to December 2017 in Lleida, Catalonia. Multimedia Appendix 1 shows the CONSORT checklist of this study.

### Target Population

Eligible population included patients with OSA (AHI ≥15) being newly diagnosed in the sleep unit of University Hospital Santa Maria, Lleida, and requiring CPAP treatment according to the Spanish Respiratory Society (SEPAR) guidelines [16]. The specific eligibility criteria were aged over 18 years; having a sufficient competence in the use of smartphones; not having been previously treated with CPAP; not having impaired lung function (overlap syndrome, obesity hypoventilation syndrome, and restrictive disorders), severe heart failure, severe chronic pathologies, psychiatric disorders, or periodic leg movements or other dyssomnias or parasomnias; and not being pregnant.

### Sample Size

Accepting an α risk of .05 and a β risk of .2 in a 2-sided test, 29 patients per study arm were needed to recognize as statistically significant a difference in compliance greater than or equal to 1 hour/day. The common SD was assumed to be 1.35, based on previous research of the group.

### Recruitment, Randomization, and Intervention

Patients were recruited in the sleep unit and randomized (1:1) to receive 6 months of either MiSAOS or usual care management. Patients in the usual care arm were managed according to the SEPAR guidelines [16]. Randomization was based on a permuted-block design with a computer random number generator and a fixed block size of 4. Patients were fitted with a mask and given a CPAP device (AirSense 10; ResMed) and a leaflet explaining how to use it. A short training session on how to use the CPAP device was also given to patients and partners in the sleep unit by a trained nurse with experience in the follow-up of CPAP-treated patients. This included a practical demonstration of how to put on the mask, and the correct management and cleaning of the tubes, mask, and humidifier. Information on how to turn the CPAP device on and off was provided by the homecare provider at the time of machine delivery. According to SEPAR recommendations, patients were visited after 1 month of treatment by the specialist nurse at the sleep unit. Information about CPAP, compliance (use of CPAP for ≥4 hours/day), residual respiratory events, and leaks was downloaded from the device. CPAP-related side effects, CPAP machine care and maintenance actions (ie, changes of mask), and the number of required additional visits or calls were recorded by the nurse.

Similarly, patients in the MiSAOS arm were fitted with a mask, a CPAP device (AirSense 10; ResMed), and given a leaflet explaining its use. Patients received the same training sessions from the same personnel as in the usual care arm. However, these patients’ CPAP devices were equipped with mobile 2G (global systems for mobile/general packet radio service [GSM/GPRS]) technology capable of sending daily information on CPAP compliance, CPAPs, mask leaks, and residual respiratory events to the MiSAOS–Oxigen salud web database. In addition, patients in the MiSAOS arm had access to an integrated platform including a website [17] and a mobile app (MiSAOS; available for both Android and iOS), benefitting from continuous monitoring and personalized feedback. Sample screenshots of the MiSAOS app showing its main functionalities and features are presented in Multimedia Appendix 2. Similarly, sample screenshots of the MiSAOS website showing some of its functionalities and features are presented in Multimedia Appendix 3. Hospital lung specialists managing these patients and the CPAP provider (Oxigen salud) also had access to the MiSAOS website that provided relevant information and decision support according to the specific role and access rights of each professional user. Finally, the cloud-based MiSAOS platform connected all the devices for data exchange and hosted an intelligent monitoring system, based on machine learning, capable of predicting the expected compliance with the therapy by a given patient, thus providing adequate feedback and proposing personalized interventions to increase compliance [15,18]. Predictions of patient’s midterm compliance were based on patient’s characteristics, such as anthropometric data and clinical information, and early compliance data. Examples of the needed information and provided outcome can be found in Multimedia Appendix 3. Based on these predictions, patients were classified into 2 groups: low compliance and medium/high compliance, and recommendations were provided based on these classes. In brief, recommendations included warnings and exhortation to do better in case of low compliance, or positive reinforcement messages in case of good compliance, highlighting the key areas to be improved regardless of the compliance. This platform was also used for the monitoring of patient compliance, prompting actions when compliance was too low.

### Data Collection

Baseline information was collected by sleep unit personnel during recruitment, regardless of the study arm. This included age; gender; socioeconomic level; Epworth Sleepiness Scale (ESS) score; EuroQoL-5D quality of life (EQ-5D); lifestyle habits (tobacco and alcohol consumption); comorbidities; use of medications; weight; height; BMI; neck, waist, and hip circumference; and BP. Variables of the sleep study were also recorded and included registration time, sleep duration, AHI, and percentage of nighttime spent with an oxygen saturation less than 90%.

At 3 and 6 months all patients, regardless of the study arm, were visited at the sleep unit. Patients were checked about progress and compliance with therapy and any problems with their CPAP machine. During these visits we collected data on treatment compliance (number of hours/day), ESS score, OSA-related symptoms, EQ-5D, BP, and anthropometric variables. Additionally, data on CPAP, residual respiratory events and leaks, CPAP-related side effects (mask allergies and skin irritations, dry mouth, congestion, runny nose, sneezing, sinusitis, nosebleeds, and discomfort), overall satisfaction with the therapy (questionnaire), CPAP machine care and maintenance actions (ie, changes of mask), and the number of any additional visits and calls required by the patient during the follow-up were collected. Finally, costs for each component, use of services, and visits were computed based on standard prices of the CPAP provider and on Catalan Health Department official data (CVE-DOGC-A-13051031-2013) [19]. Only direct costs were considered.

### Statistical Analyses

A *t* test, or an equivalent nonparametric test, or chi-square test was used for baseline bivariate analyses, depending on variables’ characteristics. Differences in the primary and secondary outcomes between the intervention and control groups at 6 months were assessed using ordinary least-squares linear models. All models were adjusted by age, and models for secondary outcomes were further adjusted by the baseline values. A 2-sided *P* value and 95% CI were used. The cost-effectiveness analysis was performed using the total costs for each arm based on intervention effectiveness (CPAP treatment compliance). A probabilistic sensitivity analysis was performed using the bootstrap method, which was represented in a cost-effectiveness plan.

The primary and secondary analyses were performed on both the intention-to-treat (ITT) and per-protocol (PP) samples. The ITT sample included all the patients who were randomized. The PP sample excluded the patients who were lost during the follow-up period. Missing data were imputed using multiple imputation consisting of chained equations, for which 10 complete databases were checked. The R package “mice” was used for these calculations. All statistical analyses and data processing procedures were performed using R software, version 3.4.4 (The R Foundation).

### Ethical Considerations

This study was approved by the Ethics Committee of Hospital Arnau de Vilanova (CEIC-1283) and all patients provided written informed consent. This project was registered in ClinicalTrials.gov (registration number NCT03116958).

## Results

**Figure 1:**
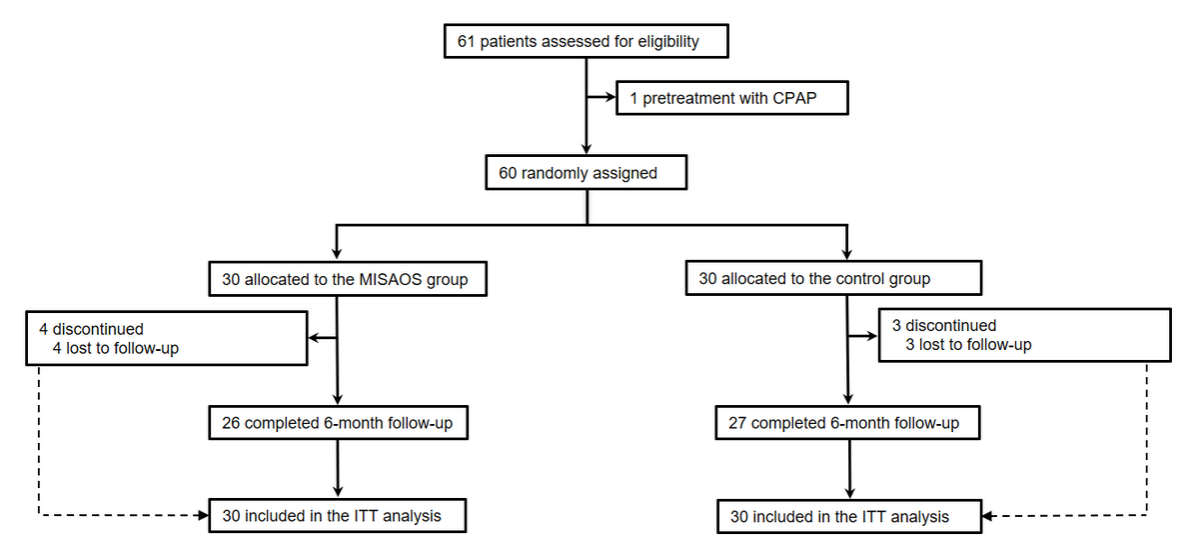
Study flowchart. CPAP: continuous positive airway pressure, ITT: intention-to-treat

A total of 60 patients were randomized to receive either MiSAOS (intervention; n=30) or usual care (control; n=30) management, and up to 53 patients completed the study (Figure 1). Patients’ baseline characteristics in both study arms are presented in Table 1, which show that only age was statistically different among groups.

Table 2 shows the primary and secondary outcomes of the trial according to an ITT analysis. After 6 months, the mean (95% CI) CPAP compliance was 4.89 (4.05-5.72) hours/day in the control group and 5.79 (5.20-6.38) hours/day in the intervention group, with an adjusted difference of 1.14 (0.04-2.23; *P*=.047) hours/day in benefit of intervention. Furthermore, the intervention arm had a higher proportion of patients with good compliance (use of CPAP for ≥4 hours/day) than the control arm (88.5% [23/26] vs 70.4% [19/27], respectively; *P*=.20), although this did not achieve statistical significance. Regarding secondary outcomes, ESS, BP, and the EQ-5D visual analog scale scores improved after 6 months of CPAP treatment in both arms, although the change in systolic BP was significantly higher in patients in the control arm than in those in the intervention arm (adjusted *P*=.04). Results on a PP approach were similar and are shown in Multimedia Appendix 4.

**Table 1:** Patients’ baseline characteristics (N=60).^a^

Characteristic	Control (n=30)	Intervention (n=30)	*P* value
Gender (male), n (%)	26 (87)	26 (87)	.99
Age (years), mean (SD)	58 (10)	52 (12)	.04
Weight (kg), mean (SD)	97 (19)	101 (23)	.42
BMI (kg/m^2^), mean (SD)	33.1 (6.4)	34.7 (7.3)	.38
Systolic blood pressure (mmHg), mean (SD)	138 (17)	142 (20)	.42
Diastolic blood pressure (mmHg), median (range)	87 (79-96)	88 (81-95)	.74
Apnea–Hypopnea Index (events/hour)	39 (25-71)	53 (35-65)	.22

^a^Data as per *t* test, or an equivalent nonparametric test, or chi-square test depending on variables’ characteristics.

**Table 2:** Differences in primary and secondary outcomes of the trial according to an intention-to-treat analysis (N=60).^a^

Differences				Control (n=30), mean (SD)		Intervention (n=30), mean (SD)		Difference, mean (95% CI)
**Primary outcome**								
	Compliance (hours/day)		4.89 (2.30)		5.79 (1.60)			
	Crude difference						0.90 (–0.16 to 1.96)	
	Adjusted difference						1.14 (0.04 to 2.23)	
**Secondary outcomes**								
	**ESS^b^ score (0-24)**							
		Baseline	10.9 (5.35)		11.1 (5.35)			
		6 months	4.90 (2.41)		5.85 (3.91)			
		Change	–5.98 (4.42)		–5.22 (4.78)			
		Crude difference					0.76 (–1.64 to 3.16)	
		Adjusted difference					1.05 (–0.51 to 2.61)	
	**Weight (kg)**							
		Baseline	97.0 (18.6)		101 (22.5)			
		6 months	98.2 (20.2)		100 (20.7)			
		Change	1.26 (7.86)		–0.95 (7.91)			
		Crude difference					–2.21 (–6.98 to 2.56)	
		Adjusted difference					–2.55 (–7.41 to 2.32)	
	**BMI (kg/m^2^)**							
		Baseline	33.3 (6.20)		34.7 (7.17)			
		6 months	34.2 (6.80)		34.8 (6.32)			
		Change	0.98 (3.26)		0.14 (3.16)			
		Crude difference					–0.84 (–2.95 to 1.27)	
		Adjusted difference					–0.82 (–2.97 to 1.32)	
	**Systolic blood pressure (mmHg)**							
		Baseline	138 (17.0)		142 (19.4)			
		6 months	131 (12.7)		138 (17.2)			
		Change	–7.02 (15.2)		–3.80 (12.7)			
		Crude difference					3.22 (–5.03 to 11.47)	
		Adjusted difference					7.81 (0.57 to 15.05)	
	**Diastolic blood pressure (mmHg)**							
		Baseline	87.7 (13.5)		90.3 (12.5)			
		6 months	81.6 (8.84)		86.8 (9.23)			
		Change	–6.13 (11.4)		–3.52 (10.6)			
		Crude difference					2.61 (–4.04 to 9.27)	
		Adjusted difference					4.52 (–0.65 to 9.69)	
	**EQ-5D^c^ HUI^d^ (0-1)**							
		Baseline	0.84 (0.22)		0.85 (0.17)			
		6 months	0.80 (0.19)		0.86 (0.20)			
		Change	–0.04 (0.17)		0.00 (0.18)			
		Crude difference					0.05 (–0.05 to 0.15)	
		Adjusted difference					0.03 (–0.06 to 0.13)	
	**EQ-5D VAS^e^ (0-10)**							
		Baseline	4.93 (3.41)		4.63 (3.55)			
		6 months	7.35 (1.71)		8.03 (1.32)			
		Change	2.42 (2.87)		3.40 (3.65)			
		Crude difference					0.98 (–0.72 to 2.69)	
		Adjusted difference					0.51 (–0.3 to 1.33)	

^a^Ordinary least-squares linear models adjusted by age and baseline value.

^b^ESS: Epworth Sleepiness Scale.

^c^EQ-5D: EuroQoL-5D quality of life.

^d^HUI: health utility index

^e^VAS: visual analog scale.

Patients’ satisfaction with the management of their illness was excellent in both study groups (Table 3). Similarly, no differences were found in the occurrence of CPAP-related side effects. Finally, all patients in the intervention group reported the MiSAOS app to be useful; 94% (16/17) of patients reported that it was easy to use, and 88% (15/17) of patients reported the willingness to continue to use it in the future (Table 3).

**Table 3:** Overall patients’ satisfaction and satisfaction with MiSAOS (N=45).^a^

Users’ satisfaction					Control (n=26)			Intervention (n=19)				*P* value	
**Overall satisfaction**													
	**The follow-up I received was sufficient to manage my health and medical needs**												
		Agrees/strongly agrees, n (%)		26 (100)			19 (100)						
		Overall score (1-7), mean (SD)		6.38 (0.80)			6.53 (0.61)				.51		
	**In general I am satisfied with the management of my illness**												
		Agrees/strongly agrees, n (%)		26 (100)		19 (100)							
		Overall score (1-7), mean (SD)		6.62 (0.57)		6.53 (0.61)				.62			
	**My contact with the hospital was sufficient**												
			Agrees/strongly agrees, n (%)	26 (100)			18 (95)						
			Overall score (1-7), mean (SD)	6.62 (0.64)			6.21 (1.62)				.31		
**Satisfaction with MiSAOS (intervention only) (n=17)**													
	**The app was useful**												
		Agrees/strongly agrees, n (%)					17 (100)						
		Overall score (1-7), mean (SD)					6.53 (0.72)						
	**The app was easy to use**												
		Agrees/strongly agrees, n (%)					16 (94)						
		Overall score (1-7), mean (SD)					6.41 (0.87)						
	**I would like to use the app every day in the future**												
		Agrees/strongly agrees, n (%)							15 (88)				
		Overall score (1-7), mean (SD)							6.35 (1.17)				

^a^The overall satisfaction questionnaire was answered by 26 controls and 19 intervention participants. The satisfaction with MiSAOS questionnaire was answered by 17 participants.

**Figure 2:**
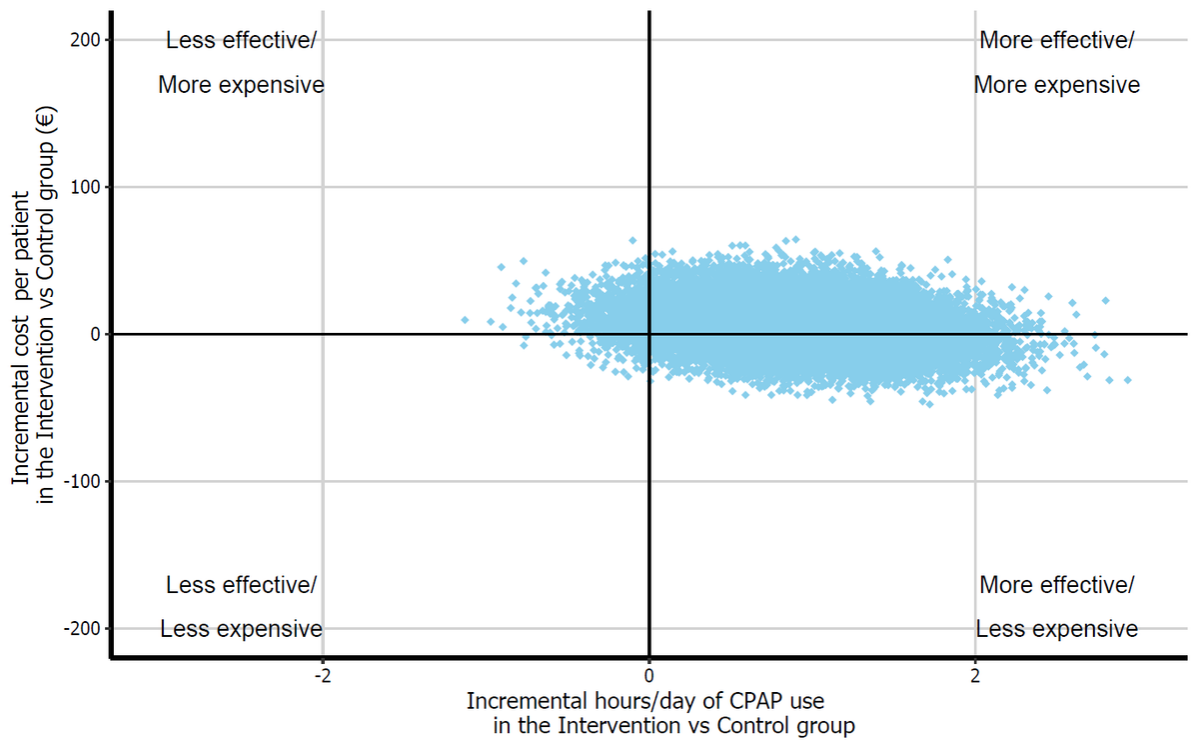
Cost-effectiveness analysis based on treatment compliance (CPAP hours/day) and total costs for each arm, performed using a bootstrap probabilistic sensitivity analysis. CPAP: continuous positive airway pressure.

Table 4 shows the costs of the intervention, costs of contacts with the CPAP provider and health system (not including the baseline, 3-month, and 6-month visits, as all patients regardless of study arm did them), and costs of any CPAP machine care and maintenance intervention actions (ie, changes of mask) during the 6 months of follow-up. The overall mean cost per patient was €90.2 (SD 53.14) (US $105.76 [SD 62.31]) in the control group and €96.2 (SD 62.13) (US $112.70 [SD 72.85]) in the intervention group, resulting in a nonsignificant cost difference between arms (*P*=.70). The main differences between arms were €49.5 (US $58.04) of the intervention costs (2G [GSM/GPRS] daily data transfer and activation and maintenance costs) in the MiSAOS arm, and the €41 (US $48.07) on sleep unit visits in the usual care arm. This overall cost, combined with the results on CPAP treatment compliance (primary outcome), demonstrated cost-effectiveness in a bootstrap-based simulation analysis (Figure 2).

**Table 4:** Within-trial intervention and follow-up costs (average cost per randomized patient; N=60).

Concept			Control (n=30), €^a^/patient, mean (SD)		Intervention (n=30), €/patient, mean (SD)		Difference, mean (95% CI)
**Intervention costs^b^**							
	2G (GSM^c^/GPRS^d^) daily data transfer	0 (0)		41.5 (0)		–41.5 (—)	
	Activation and maintenance	0 (0)		8 (0)		–8 (—)	
**Follow-up costs**							
	Sleep unit visits and consultations^e^	41 (0)		0 (0)		41 (—)	
	CPAP^f^ provider visits and consultations^g^	9.7 (8.9)		10.0 (10.8)		–0.33 (–5.5 to 4.8)	
**Changes in CPAP device components^h^**							
	ResMed Mirage Quattro	12.5 (28.4)		10.0 (32.6)		2.5 (–13.3 to 18.3)	
	ResMed Mirage FX	0.8 (4.4)		3.2 (8.3)		–2.4 (–5.9 to 1.1)	
	ResMed Mirage Micro	0 (0)		1.1 (4.1)		–1.07 (–2.6 to 0.4)	
	ResMed Swift FX	1.5 (8.2)		4.5 (13.7)		–3 (–8.9 to 2.9)	
	ResMed Airfit P10 (without head-gear)	0 (0)		1.3 (7.3)		–1.33 (–4.1 to 1.4)	
	Philips Respironics Nuance gel	0 (0)		2.45 (13.4)		–2.45 (–7.5 to 2.6)	
	ResMed Airfit F10	15.0 (30.5)		10.0 (25.9)		5 (–9.6 to 19.6)	
	ResMed Airfit P10 (with head-gear)	1.50 (8.22)		0 (0)		1.5 (–1.6 to 4.6)	
	SleepNet IQ	2.20 (8.86)		0 (0)		2.2 (–1.1 to 5.5)	
	SleepNet Ascend	0 (0)		0.8 (4.4)		–0.8 (–2.4 to 0.8)	
	Philips Respironics Amara View	5.00 (15.3)		3.3 (18.3)		1.66 (–7.0 to 10.4)	
	Philips Respironics Comfort Gel Blue	1.00 (5.48)		0 (0)		1 (–1.0 to 3.0)	
	Total	90.2 (53.1)		96.2 (62.1)		–6.0 (–35.9 to 23.9)	

^a^€1 = US $1.17.

^b^Estimated costs supplied by the CPAP provider: 2G (GSM/GPRS) daily data transfer (€83 [US $97.32]/year); activation and maintenance (€16 [US $18.76]/year).

^c^GSM: global systems for mobile.

^d^GPRS: general packet radio service.

^e^Not including the baseline, 3-month, and 6-month visits, as all patients did them regardless of study arm. Costs based on the Catalan Institute of Health (CVE-DOGC-A-13051031-2013): sleep unit visits and consultations (€41 [US $48.07]/contact).

^f^CPAP: continuous positive airway pressure.

^g^Commercial costs supplied by the CPAP provider: CPAP provider visits and consultations (€10 [US $11.73]/contact).

^h^Commercial costs supplied by the CPAP provider: ResMed Mirage Quattro (€75 [US $87.94]/unit); ResMed Mirage FX (€24 [US $28.14]/unit); ResMed Mirage Micro (€16 [US $18.76]/unit); ResMed Swift FX (€45 [US $52.76]/unit); ResMed Airfit P10 (without headgear) (€40 [US $46.90]/unit); Philips Respironics Nuance gel (€73.5 [US $86.18]/unit); ResMed Airfit F10 (€75 [US $87.94]/unit); ResMed Airfit P10 (with head-gear) (€45 [US $52.76]/unit); SleepNet IQ (€22 [US $25.80]/unit); SleepNet Ascend (€24 [US $28.14]/unit); Philips Respironics Amara View (€50 [US $58.63]/unit); Philips Respironics Comfort Gel Blue (€30 [US $35.18]/unit).

## Discussion

### Principal Findings

This study is the first randomized controlled clinical trial assessing the effectiveness and cost-effectiveness of a machine learning–based intelligent monitoring system aiming to improve CPAP compliance in patients with OSA. The MiSAOS intelligent monitoring system, based on early compliance detection, compliance prediction, and rule-based recommendations, was compared with usual care in the region of Lleida, showing a mean increase of 1.14 hours in daily compliance with no substantial differences in direct costs and an excellent patient satisfaction. This novel management system proved to be cost-effective and thus a viable option for the management of patients with OSA treated with CPAP.

### Strengths and Limitations

This study has several strengths, including the (1) use of the same CPAP devices in both study arms; (2) use of an intelligent monitoring system model, based on early compliance detection, machine learning–based compliance prediction, and rule-based recommendations; (3) inclusion of continuous patient feedback through an app; (4) measurement of a broad range of effect measures (ie, compliance, changes in symptoms, and changes in quality of life); (5) assessment of patient comfort and satisfaction; and (6) inclusion of cost and cost-effectiveness analyses. Nevertheless, there are also some limitations to be acknowledged: (1) the slight infraestimation of the required number of study participants limited the statistical power of some of the between-arm comparisons, although this did not affect the results on the primary outcome and cost-effectiveness analysis; (2) the assessment of patient satisfaction was performed using a nonvalidated questionnaire; (3) the exclusion of patients with severe chronic pathologies and other dyssomnias or parasomnias could limit the generalizability of our results, although the included patients would be the ideal target for eHealth interventions as more complex patients could require a close follow-up in the sleep units; (4) the results of cost analyses are highly dependent on the characteristics of the health care setting in which they are conducted and, thus, extrapolation of the results to different settings should be done cautiously; and (5) the follow-up period does not allow the extrapolation of results to the long term.

### Comparison With Existing Literature

Patients experiencing the MiSAOS intelligent monitoring system showed a mean increase of 1.14 hours in daily CPAP compliance when compared with patients in usual care. This result is more positive than the mean (95% CI) increase of 0.54 (0.29-0.79) hours reported by Aardoom et al [14] in a 2020 meta-analysis including 18 studies with eHealth interventions. Other recent studies exploring advanced monitoring systems have shown similarly inferior results, for instance, Pépin et al [20] reported a 0.53 hours’ increase in CPAP compliance in patients with OSA with high cardiovascular risk using a multimodal telemonitoring intervention. Interestingly, granting patients an easy access to their compliance data has shown successful results in terms of increasing patient compliance [21-23]. Therefore, the combination of an intelligent machine learning–based monitoring system with the empowerment of patients, based on access to daily compliance and personalized feedback through an app, could represent a significant step forward in the promotion of CPAP compliance.

The impact of the CPAP treatment on secondary outcomes in the MiSAOS intervention was very similar to that achieved in usual care and reports in previous literature [12,20]. Sleepiness, overall quality of life, and BP improvements after 6 months of follow-up were similar in both study arms. The only difference between study arms was the significantly higher decrease in systolic BP in patients having usual care, which could be easily explained by the baseline differences in BP between study arms.

Patient’s comfort and satisfaction are key drivers of compliance with CPAP treatment in the long term [9]. On the one hand, regarding comfort, the number of side effects in both arms was very similar. On the other hand, it must be noticed that in telemedicine interventions patients’ satisfaction is usually similar to or lower than that of usual care [12], with privacy concerns being the main reported issue [24,25]. In this trial, all patients reported excellent satisfaction with their management, regardless of the study arm. Moreover, patients in the intervention group considered the MiSAOS app as useful and easy to use and reported their willingness to keep using it in the future. These results are better than those obtained in telemonitoring interventions in the same setting (Lleida, Spain) but not providing any direct feedback to the patients [25] and confirm that patient empowerment has a direct impact on patient satisfaction. Finally, potential issues on data privacy had no impact on the current trial results, in contrast to previous research [25], and in line with other interventions providing the patients with regular feedback on compliance [21].

A key aspect of any new management strategy is the cost of the intervention and its cost-effectiveness. In this study, the analysis of costs and cost-effectiveness showed that the MiSAOS intervention had an overall cost similar to that of usual care while providing better results in terms of treatment compliance, thus demonstrating cost-effectiveness. This result is in contrast to previous cost-effectiveness trials of telemonitoring interventions for CPAP-treated patients in Spain, where cost-effectiveness was demonstrated because of an overall reduction in costs and no significant differences in effectiveness were found [25,26]. Similarly, telemedicine platforms with automated functions to provide education or accountability have already shown cost-effectiveness in sleep medicine [23]. This suggests that the addition of machine learning data–processing functionalities together with the empowerment of patients by means of direct feedback could tip the scales toward significant increases in compliance and boost the cost-effectiveness of already existing telemonitoring interventions. Moreover, it should be noticed that a key factor of telemonitoring is the reduction in the number or duration of follow-up visits, which was quantified by Anttalainen et al [27], reporting a saving of 19 minutes in nursing time when comparing telemonitoring with usual care in the habituation phase of CPAP treatment (4 weeks) [27], and should be sufficient to mitigate the costs of telemonitoring.

### Implications for Future Clinical Practice

As previously stated, the main barriers for the large-scale implementation of a novel management intervention are its costs and cost-effectiveness. In the optimal scenario, a novel management strategy should be cheaper than usual care while providing better results. The MiSAOS model has shown the potential to generate better results than usual care in terms of compliance. However, it was not cheaper than usual care. It is worth mentioning that a big proportion of the intervention’s cost corresponded to the use of a 2G (GSM/GPRS) system for daily CPAP compliance data transfer. This technology could be easily replaced by a secure wireless connection to the patients’ home Wi-Fi network, which would represent a huge saving and further boost cost-effectiveness. Even in rural areas such as Lleida, this scenario is rapidly becoming a reality and most homes have a suitable Wi-Fi network.

### Conclusion

The use of a machine learning–based intelligent monitoring system increased daily compliance, reported an excellent patient satisfaction similar to that reported in usual care, and did not incur in a substantial increase in costs, thus proving cost-effectiveness. This study supports the implementation of intelligent eHealth frameworks for the management of patients with OSA treated by CPAP and confirms the value of patients’ empowerment in the management of chronic diseases.

## Appendix

Multimedia Appendix 1 CONSORT-eHealth checklist (V 1.6.1).

Multimedia Appendix 2 Screenshots of the MiSAOS app showing from left to right and top to bottom: (i) loading screen with the app’s name; (ii) last week’s overall CPAP treatment performance summary, including an overall numeric score (0-10) together with a summary smiley, average compliance in hours, average air leaks, average residual apnea-hypopnea index (AHI), specific compliances for the last 7 days, and a summary text including reinforcements and tips to improve the overall rating in upcoming weeks (in this case, a positive reinforcement message for and a tip regarding air-leaks and hours of use); (iii) last week’s overall CPAP treatment performance summary with focus on air-leaks, including last 14 days air-leak information and additional details; (iv) patient and device’s summary information including technical details on the CPAP treatment characteristics; (v) main achievements summary, including a ranking of the patient’s performance compared to other CPAP users in the region and challenges’ progression such as good compliance streaks; and, (vi) sample information and training screen, in this case explaining the basics of obstructive sleep apnea pathophysiology.

Multimedia Appendix 3 Screenshots of the MiSAOS website showing from top to bottom: (i) registry of events and actions taken in relation to a given patient, including date, action details and comments; (ii) example of data collection for the feed of the predictive algorithms; (iii) sample prediction provided by MiSAOS intelligent algorithms, in this case predicting 6-month compliance based on early compliance and information such as the one collected in the previous screenshot; and, (iv) example of available training material tackling the most common issues and doubts of patients (in addition to videos there are also manuals, FAQs, and tips).

Multimedia Appendix 4 Differences in primary and secondary outcomes of the trial according to a per-protocol analysis.

## Abbreviations

AHI: Apnea–Hypopnea Index
BP: blood pressure
CPAP: continuous positive airway pressure
ESS: Epworth Sleepiness Scale
ITT: intention-to-treat
OSA: obstructive sleep apnea
PP: per protocol
SEPAR: Spanish Respiratory Society
