# Supplementary figures and images for: Management and Treatment of Patients With Obstructive Sleep Apnea Using an Intelligent Monitoring System Based on Machine Learning Aiming to Improve Continuous Positive Airway Pressure Treatment Compliance: Randomized Controlled Trial
Source: J Med Internet Res. 2021 Oct 18;23(10):e24072. doi: 10.2196/24072 (PMC8561405; doi:10.2196/24072)

# Screenshots of the MiSAOS app


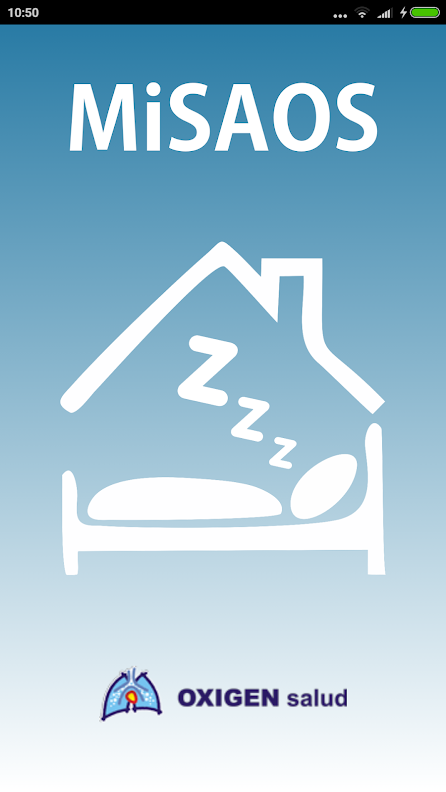

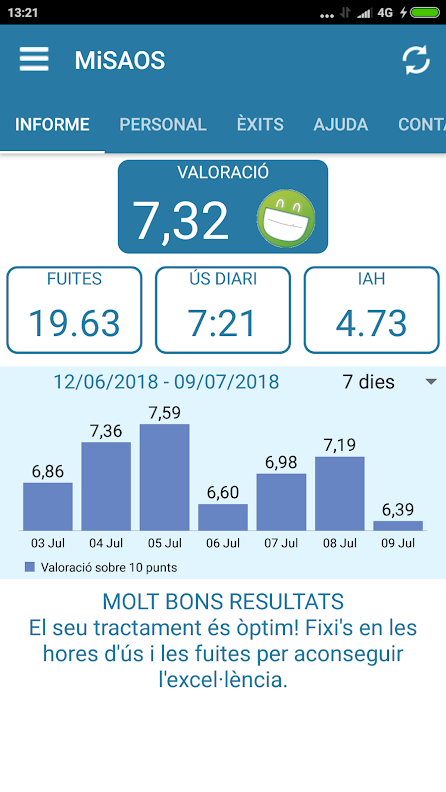

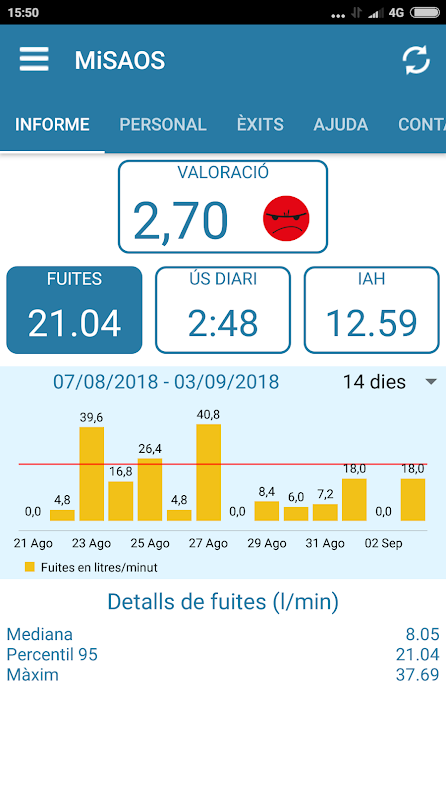


(i) (ii) (iii)


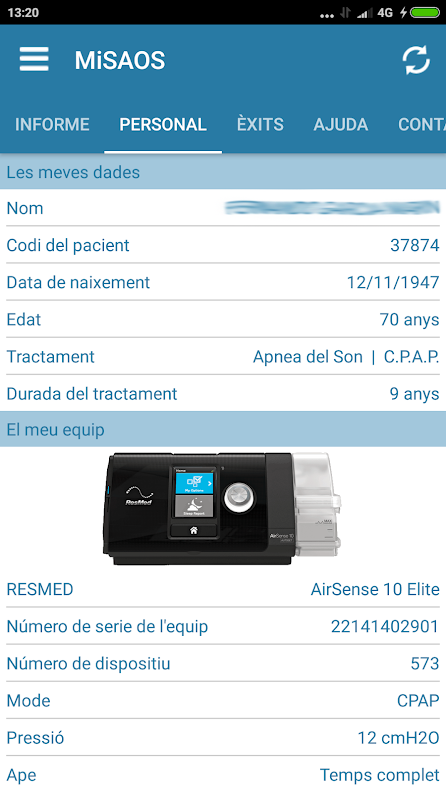

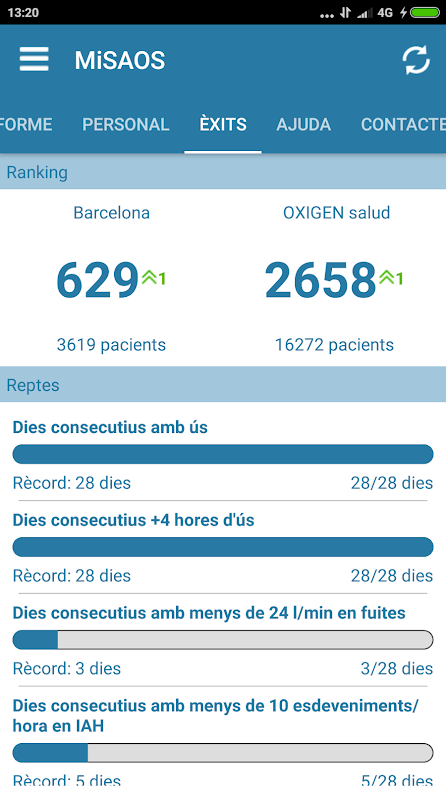

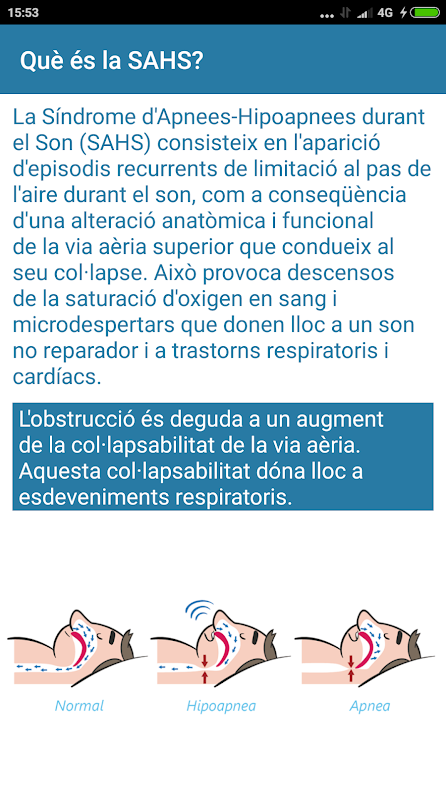


(iv) (v) (vi)

Supplement: Multimedia Appendix 2 [file jmir_v23i10e24072_app2.docx]

# Screenshots of the MiSAOS website


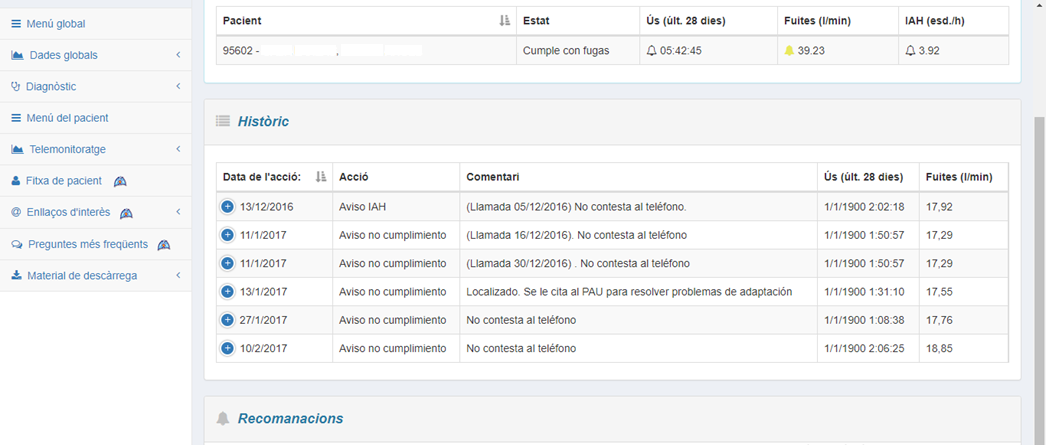


(i)


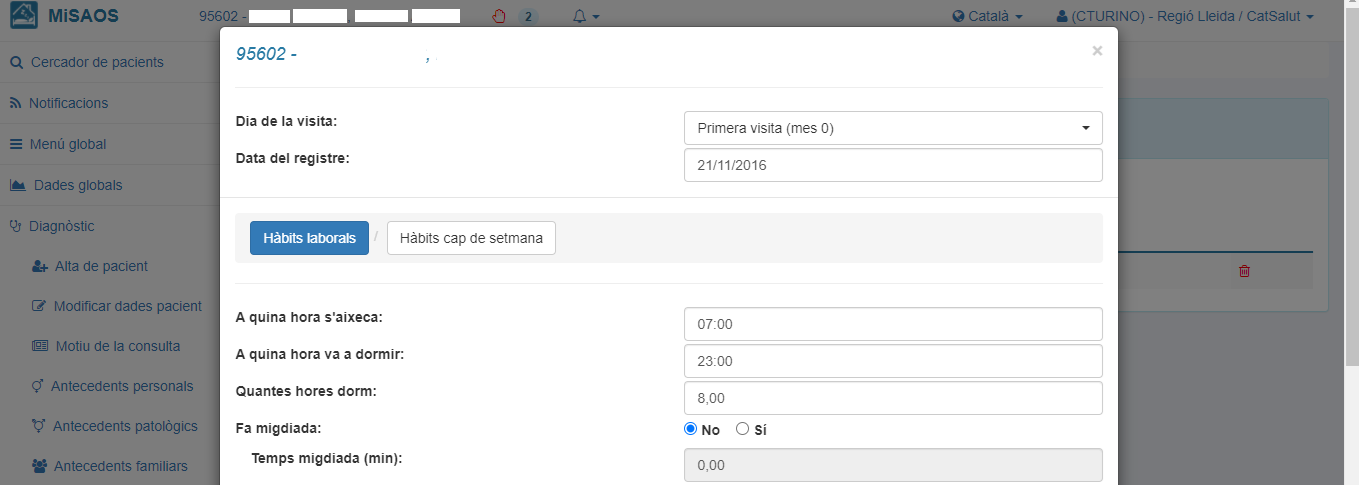


(ii)


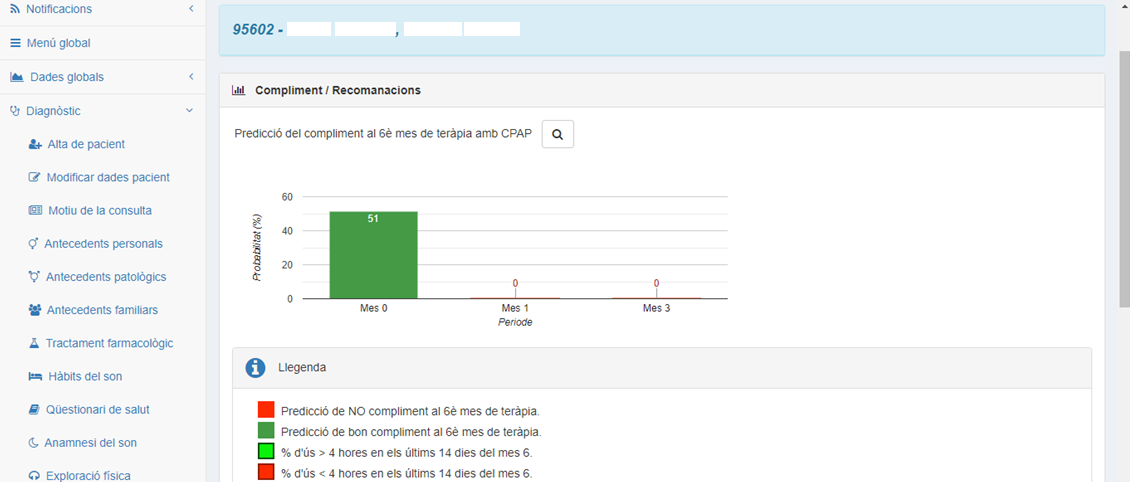


(iii)


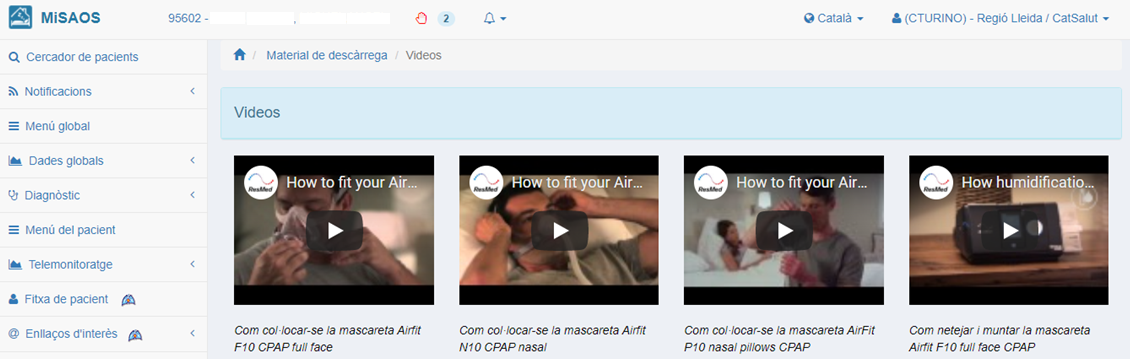


(iv)

Supplement: Multimedia Appendix 3 [file jmir_v23i10e24072_app3.docx]
