# Supplementary material for: Management and Treatment of Patients With Obstructive Sleep Apnea Using an Intelligent Monitoring System Based on Machine Learning Aiming to Improve Continuous Positive Airway Pressure Treatment Compliance: Randomized Controlled Trial
Source: J Med Internet Res. 2021 Oct 18;23(10):e24072. doi: 10.2196/24072 (PMC8561405; doi:10.2196/24072)
Supplement: Multimedia Appendix 4 [file jmir_v23i10e24072_app4.docx]

| **Table S1**. Differences in primary and secondary outcomes of the trial according to a per-protocol analysis. | | | | | | |
| --- | --- | --- | --- | --- | --- | --- |
|  | **Control** | **Intervention** | **Difference** | | | |
|  | **(n = 30)** | **(n=30)** | **Crude** | | **Adjusted** | |
|  | **Mean (95% CI)** | **Mean (95% CI)** | **Mean (95% CI)** | ***P*** | **Mean (95% CI)** | ***P*** |
| **Primary Outcome** |  |  |  |  |  |  |
| Compliance (hours/day) | 4.95 (2.33) | 5.68 (1.62) | 0.73 (-0.39 to 1.84) | 0.196 | 1.01 (-0.14 to 2.16) | 0.085 |
| **Secondary Outcomes** |  |  |  |  |  |  |
| ESS (0-24) |  |  |  |  |  |  |
| Baseline | 10.05 (5.28) | 11.69 (5.28) |  |  |  |  |
| 6 months | 4.73 (2.44) | 6.04 (4.12) |  |  |  |  |
| Change | -5.32 (4.23) | -5.65 (4.78) | -0.33 (-2.84 to 2.19) | 0.794 | -1.68 (-6.08 to 2.71) | 0.443 |
| Weight (kg) |  |  |  |  |  |  |
| Baseline | 98.33 (16.00) | 100.99 (21.67) |  |  |  |  |
| 6 months | 98.58 (17.81) | 99.53 (19.51) |  |  |  |  |
| Change | 0.25 (5.94) | -1.46 (7.58) | -1.71 (-5.89 to 2.48) | 0.415 | -0.25 (-1.87 to 1.37) | 0.755 |
| BMI (kg/m^2^) |  |  |  |  |  |  |
| Baseline | 33.62 (5.52) | 34.82 (6.38) |  |  |  |  |
| 6 months | 33.93 (6.10) | 34.67 (5.36) |  |  |  |  |
| Change | 0.31 (2.14) | -0.15 (2.88) | -0.45 (-2.01 to 1.11) | 0.561 | 0.86 (-0.86 to 2.58) | 0.32 |
| Systolic BP (mm Hg) |  |  |  |  |  |  |
| Baseline | 137.77 (17.27) | 142.20 (20.02) |  |  |  |  |
| 6 months | 132.22 (12.20) | 140.90 (18.08) |  |  |  |  |
| Change | -7.67 (15.13) | -6.18 (11.22) | 1.49 (-6.97 to 9.95) | 0.724 | 7.81 (0.97 to 14.65) | 0.026 |
| Diastolic BP (mm Hg) |  |  |  |  |  |  |
| Baseline | 89.24 (14.01) | 92.14 (13.42) |  |  |  |  |
| 6 months | 82.20 (7.92) | 86.53 (9.78) |  |  |  |  |
| Change | -7.04 (11.37) | -5.61 (8.24) | 1.43 (-5.01 to 7.88) | 0.656 | 3.52 (-0.94 to 7.98) | 0.118 |
| EQ-5D HUI (0-1) |  |  |  |  |  |  |
| Baseline | 0.82 (0.24) | 0.85 (0.18) |  |  |  |  |
| 6 months | 0.79 (0.19) | 0.85 (0.21) |  |  |  |  |
| Change | -0.04 (0.18) | 0.01 (0.18) | 0.04 (-0.06 to 0.15) | 0.398 | 0.03 (-0.06 to 0.13) | 0.475 |
| EQ-5D VAS (0-10) |  |  |  |  |  |  |
| Baseline | 4.96 (3.52) | 4.42 (3.55) |  |  |  |  |
| 6 months | 7.38 (1.79) | 8.00 (1.36) |  |  |  |  |
| Change | 2.42 (2.91) | 3.58 (3.71) | 1.15 (-0.7 to 3.01) | 0.218 | 0.43 (-0.42 to 1.29) | 0.309 |
| Ordinary least-squares linear models adjusted by age and baseline value. ESS: Epworth sleepiness scale; BMI: body mass index; BP: blood pressure; EQ-5D: EuroQoL-5D quality of life; HUI: health utility index; VAS: visual analog scale. | | | | | | |
